# *Tempranillo* Grape Extract in Transfersomes: A Nanoproduct with Antioxidant Activity

**DOI:** 10.3390/nano12050746

**Published:** 2022-02-23

**Authors:** Carlos Asensio-Regalado, Rosa María Alonso-Salces, Blanca Gallo, Luis A. Berrueta, Benedetta Era, Francesca Pintus, Carla Caddeo

**Affiliations:** 1 Department of Analytical Chemistry, University of the Basque Country UPV/EHU, P.O. Box 644, 48080 Bilbao, Spain; carlos.asensio@ehu.eus (C.A.-R.); blanca.gallo@ehu.eus (B.G.); luisangel.berrueta@ehu.eus (L.A.B.); 2 Consejo Nacional de Investigaciones Científicas y Técnicas (CONICET), CIAS-IIPROSAM, Facultad de Ciencias Exactas y Naturales, Universidad Nacional de Mar del Plata, Funes 3350, Mar de Plata 7600, Argentina; rosamaria.alonsosalces@gmail.com; 3 Department of Scienze della Vita e dell’Ambiente, Sezione Biomedica, University of Cagliari, SS 554-bivio per Sestu, 09042 Cagliari, Italy; era@unica.it (B.E.); fpintus@unica.it (F.P.); 4 Department of Scienze della Vita e dell’Ambiente, Sezione di Scienze del Farmaco, University of Cagliari, Via Ospedale 72, 09124 Cagliari, Italy

**Keywords:** grape pomace extract, phospholipid vesicles, skin delivery, skin cells, antioxidant

## Abstract

Polyphenols are gaining increasing interest due to their beneficial properties to human health. Grape pomace, the by-product of wine production, is a source of these bioactive compounds. An extract from *Tempranillo* grape pomace was obtained and characterized qualitatively and quantitatively. The major components found were anthocyanins, flavan-3-ols, and flavonols. To improve the bioavailability of these compounds, the extract was formulated in phospholipid vesicles, namely transfersomes. Spherical unilamellar vesicles around 100 nm each were obtained. The antioxidant activity of both the extract and the transfersomes was evaluated by using colorimetric assays (i.e., DPPH, FRAP, and Folin–Ciocalteu). The cells’ viability and the antioxidant activity were assessed in keratinocytes. The results showed that the extract and the transfersomes had no cytotoxic effects and exerted remarkable antioxidant activity, which was more evident in a vesicle formulation. These findings highlighted the potential of the *Tempranillo* grape pomace extract and the efficacy of the incorporation into phospholipid vesicles.

## 1. Introduction

The normal functioning of the body involves several biological reactions in cells and tissues that often generate species with unpaired electrons called free radicals, such as reactive oxygen species (ROS). These compounds are usually balanced by endogenous mechanisms, but an excessive accumulation can lead to oxidative stress in cells [1,2]. In particular, the skin is continuously exposed to chemical, mechanical, and physical stress, which leads to an excess of ROS and other free radicals [3]. The accumulation of these species has been linked to the development of chronic inflammatory conditions and tumor initiation and promotion. An external intake of antioxidants has demonstrated an effect on counterbalancing these processes and restoring physiological conditions. Naturally occurring antioxidants include polyphenols, which are present in fruits and plants and have been demonstrated to have antioxidant, antiradical, antimicrobial, and anti-inflammatory properties that help protect and prevent chronic diseases and cancer [4].

The grape is one of the fruits with the highest content of phenolic antioxidants. Its main components include anthocyanins, catechins, procyanidins, and tannins [5]. The grape has been employed for winemaking since ancient times, and wine consumption has been associated with an improvement in cardiovascular conditions [6,7] amongst other benefits. Grape skins are rich in anthocyanins, as well as other bioactive compounds [8]. Due to an incomplete extraction during winemaking, these compounds largely accumulate in the pomace, mostly composed of seeds, skins, and stems [9,10]. These residues represent a waste problem, but also an opportunity to obtain a sustainable and low-cost source of antioxidants. Various industrial sectors, such as pharmaceuticals, foods, and cosmetics, can benefit from the valorization of wine-processing materials [5,11,12].

To maximize the potential of the grape’s pomace, extraction method is an important factor to consider. The efficacy and commercial feasibility of the extract depend highly on the use of convenient, inexpensive, and eco sustainable procedures that ensure the highest yield and quality of active compounds of the extract [13]. For this reason, in this study, the extraction of bioactive compounds from grape pomace was performed by using a simple procedure that involved the use of an ethanol/water mixture.

However, polyphenolic compounds have shown some limitations, such as a low in vivo bioavailability and easy degradation [14]. Their topical application has some potential advantages, such as the avoidance of hepatic first-pass metabolism and gastric degradation, a larger surface area for absorption, low proteolytic activity levels, and ease of accessibility [15].

In an attempt to improve the applicability of phenolic compounds, different innovative strategies have been used, including solid dispersions [16], nanosuspensions [17], microemulsions [18], solid lipid nanoparticles [19], and liposomes [20], the latter being one of the most successful. Over the last years, liposomes have been the target of reformulating studies aimed at producing vesicles capable of delivering drugs to the deeper skin layers. A number of additives have been explored in combination with conventional components of liposomes, producing new classes of vesicles, such as transfersomes. Transfersomes are composed of phospholipids and an edge activator, which is a membrane-softening agent (e.g., Tween 80, Span 80, and sodium cholate) that makes the vesicle ultra-deformable [21,22]. Unlike conventional liposomes, when transfersomes reach skin pores, they are capable of changing their membrane flexibility and passing through the skin pores spontaneously, thus promoting the accumulation of a payload in the dermis. A number of transfersome-based formulations are currently being assessed at different stages of clinical trials [23].

In light of these considerations, the aim of this work was to develop, optimize, and characterize a vesicle formulation that increases the bioavailability of the bioactive compounds of a grape pomace extract to be applied on the skin. An extract of *Tempranillo* grape pomace was produced, characterized by liquid chromatography coupled to UV-visible spectrophotometry and mass spectrometry and formulated in transfersomes. The vesicles were characterized by morphology, size, surface charge, storage stability, and entrapment efficiency and tested for antioxidant activity in vitro and in cell cultures.

## 2. Materials and Methods

### 2.1. Materials

Phospholipon 90G (P90G) was supplied by Lipoid GmbH (Ludwigshafen, Germany). Polyoxyethylene (20) sorbitan mono-oleate (Tween 80) was supplied by Galeno (Carmignano, Prato, Italy). Standard malvidin-3-*O*-glucoside (Mv-3-*O*-glc) was purchased from Extrasynthèse (Genay, France). Acetonitrile 190 was purchased from Teknokroma (Barcelona, Spain). Trifluoroacetic acid (TFA for spectroscopy, Uvasol^®^), Folin–Ciocalteu’s reagent, 2,2-diphenyl-1-picrylhydrazyl (DPPH), 6-hydroxy-2,5,7,8-tetramethylchroman-2-carboxylic acid (Trolox), 2,4,6-tris(pyridin-2-yl)-1,3,5-triazine (TPTZ), and other reagents were supplied by Merck/Sigma-Aldrich (Darmstadt, Germany) unless otherwise specified.

### 2.2. Grape Pomace Extract Preparation

**Figure 1:**
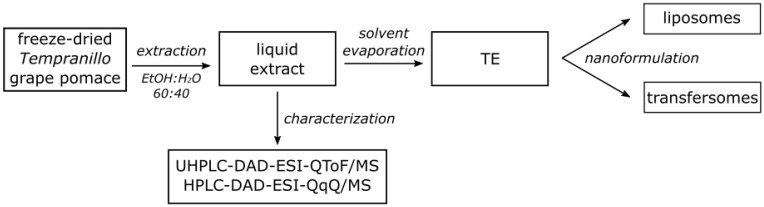
Scheme of extraction, characterization and nanoformulation of the extract.

A pomace from the *Tempranillo* red grape, a Spanish autochthonous cultivar, was supplied by Bodegas Faustino winery (Oyón, Spain). The pomace was stored at −20 °C. Prior to an extraction, the pomace was freeze-dried and ground to a coarse powder using Ø11 mm stainless steel balls in a rotary agitator. An aliquot of the freeze-dried powder (25 g) was soaked in 500 mL of ethanol/water (60:40, *v*/*v*), sonicated in an ultrasonic bath for 5 min (40 kHz; r.t.), and centrifuged (20 min, 8000 rpm, 4 °C) to collect the supernatant. Phenolic compounds in the *Tempranillo* grape pomace extract were identified by using ultra-high-performance liquid chromatography (UHPLC)—coupled to a diode array detector (DAD) and an electrospray-ionization (ESI) quadrupole time-of-flight mass spectrometer (QToF/MS)—and anthocyanins were quantified by using high-performance liquid chromatography (HPLC) coupled to a DAD and ESI triple-quadrupole mass spectrometer (QqQ/MS). Then, the ethanol of the extract was evaporated at 30 °C under vacuum prior to freeze-drying. The yield was calculated as the ratio of freeze-dried extract weight/starting freeze-dried material weight in percentages. The obtained *Tempranillo* extract (TE) was used for preparing the nanoformulations (Section 2.5; Figure 1).

### 2.3. Identification of Phenolic Compounds by Using UHPLC-DAD-ESI-QToF/MS

The phenolic profile of the extract was characterized by using a UHPLC-DAD-ESI-QToF/MS, an ACQUITY UPLC^TM^ system coupled to a DAD and a SYNAPT^TM^ G2 HDMS (Waters, Milford, MA, USA). The separation was carried out using a reversed-phase ACQUITY UPLC BEH C18 column (100 × 2.1 mm, 1.7 µm) with a pre-column of the same material (VanGuard^TM^) (Waters, Milford, MA, USA) and a method described by Garrido et al. [24] with minor modifications. The separation was carried out using 0.1% (*v*/*v*) of acetic acid in water and 0.1% (*v*/*v*) of acetic acid in methanol as mobile phases. The injection volume was 5.0 µL. Flavan-3-ols were recorded at 280 nm, as were hydroxycinnamic acids at 320 nm and flavonols at 370 nm. Mass spectral data were recorded in positive and negative ion modes.

### 2.4. Analysis of Anthocyanins by Using HPLC-DAD-ESI-QqQ/MS

The anthocyanins in the extract were determined by using an HPLC-DAD-ESI-QqQ/MS, an Alliance 2695 with a DAD, and a Micromass Quattro micro^TM^-API tandem quadrupole system with a Z-spray ESI source (Waters) working in positive ion mode. A reversed-phase Luna C18 column (150 × 4.6 mm, 3 µm; Phenomenex, Torrance, CA, USA) with a pre-column of the same material was used. Mobile phases consisting of aqueous TFA (0.5% v; A) and acetonitrile (B) were delivered at a flow rate of 0.8 mL/min. A gradient program was used: 0–15 min linear gradient at 12 to 15% B; 15–25 min isocratic elution at 15% B; 25–40 min linear gradient at 15 to 25% B; 40–50 min linear gradient at 25 to 30% B; 50–55 min linear gradient at 30 to 100% B; and 55–60 min isocratic elution at 100% B. An aliquot of 50 μL of extract was injected after a filtration through an Acrodisc^®^ filter with a PTFE membrane (0.45 µm, ø 13 mm, Pall Corporation, NY, USA). The injector and column temperatures were 4 °C and 30 °C, respectively. The UV-vis spectra of the chromatographic peaks were registered each second in a 250–600 nm range. The mass spectrometer used nitrogen at 300 °C and 450 L/h as a desolvation gas. The capillary potential was 3.2 kV in positive ion mode, and 120 °C was the source-block temperature. Anthocyanins were quantified at 530 nm. Mv-3-*O*-glc, the major anthocyanin present in the extract, was used as a standard for the external calibration in the range of 0.5–200 mg/L. It was prepared with a stock solution in methanol with 0.1% HCl (*v*/*v*) for better stability. The concentrations of the detected compounds were expressed as equivalent concentrations of Mv-3-*O*-glc.

### 2.5. Vesicle Preparation and Characterization

To produce transfersomes, TE (10 mg/mL), P90G (120 mg/mL), and Tween 80 (10 mg/mL) were dispersed in water and sonicated (5 cycles of 5 s on/2 s off + 3 cycles 3 s on/2 s off; 13 µm of probe amplitude) with a Soniprep 150 (MSE Crowley, London, UK). TE liposomes (i.e., without Tween 80) and empty transfersomes and liposomes (i.e., without TE) were prepared for an appropriate comparison.

Cryogenic-transmission electron microscopy (cryo-TEM) was employed to examine the formations and morphologies of the vesicles. Three μL of the dispersion was placed on a glow-discharged 300-mesh Quantifoil grid and plunge frozen into liquid ethane in an FEI Vitrobot Mark IV (Eindhoven, The Netherlands). The frozen grid was transferred first to a 626 DH Single Tilt Cryo-Holder (Gatan, France), where it was kept below −180 °C, and then to a TECNAI G2 20 TWIN (FEI), operating at a 200 KeV accelerating voltage in a bright-field low-dose image mode.

Dynamic and electrophoretic light scattering techniques were used to measure the average diameters, polydispersity indexes and zeta potentials of the vesicles. The dispersions were diluted with water (1:30, *v*/*v*) and analyzed using a Zetasizer nano-ZS (Malvern Panalytical, Worcestershire, UK).

The above parameters were monitored over two months at 4 ± 2 °C to evaluate the storage stabilities of the formulations.

Dialysis was performed to remove the non-incorporated extract constituents from the vesicle dispersions. One mL of sample was loaded into Spectra/Por^®^ tubing (12,000–14,000 Da MWCO; Spectrum, DG Breda, The Netherlands) and kept in water (2 L) under gentle stirring for 2 h. Non-dialyzed and dialyzed vesicles were disrupted by diluting (1:50, *v*/*v*) them with methanol:water (40:60, *v*/*v*) and analyzed by using HPLC-DAD-ESI-QqQ/MS to determine the amounts of anthocyanins. The entrapment efficiency (EE) was calculated as the percentages of the anthocyanins detected in dialyzed vs. non-dialyzed samples.

### 2.6. Antioxidant Assays

The total phenolic contents of the TE methanolic solution and vesicle dispersions were determined by using the Folin–Ciocalteu method with minor modifications [25]. The vesicle dispersions were sonicated (6 cycles with 10 s on and 2 s off) to disrupt the vesicles and free the components from the TE. An amount of 10 µL of each sample was mixed with 50 µL of Folin–Ciocalteu’s reagent (2 N) and 790 µL of water. After 1 min, 150 µL of a 20% aqueous solution of sodium carbonate was added. After 45 min of an incubation at r.t. in the dark, the samples were centrifuged, and the absorbance of the supernatant was read at 750 nm. The TE methanolic solution was processed according to the above procedure, but without the sonication and centrifugation steps. The total phenolic content was expressed as µg of gallic acid equivalents (GAE)/mL of solution.

The antioxidant activity of the TE methanolic solution and the empty and TE transfersomes was assessed by means of the DPPH assay. Forty µL of each sample was mixed with 2 mL of a 25 µM DPPH methanolic solution. After 30 min of incubation at room temperature in the dark, the absorbance (A) was recorded at 517 nm. The discoloration of the DPPH solution corresponded to a decrease in absorbance, which was correlated to the antioxidant power and the concentration of the sample. The antioxidant activity (AA) was calculated according to Equation (1):

AA = ((A_DPPH_ − A_sample_)/A_DPPH_) × 100 (1)

The antioxidant activity was expressed also as Trolox equivalents. The µg Trolox equivalents/mL solution were calculated using a calibration curve (Trolox concentration range: 0–500 µg/mL).

The antioxidant activity of the TE methanolic solution and the empty and TE transfersomes was assessed by using the FRAP (ferric-reducing antioxidant power) assay, which is based on a reduction of Fe^3+^-TPTZ to Fe^2+^-TPTZ that causes an increase in absorption [26]. Twenty µL of each sample was mixed with 2 mL of the TPTZ-ferric solution. After 4 min of incubation at room temperature in the dark, the absorbance was read at 593 nm. The results, expressed as µg Fe^2+^ equivalents/mL of solution, were calculated using a calibration curve (FeSO_4_ concentration range: 0–1200 µg/mL).

### 2.7. Cell Culture and Intracellular ROS Levels

Human skin keratinocytes (HaCaT; CLS–Cell Lines Service, Eppelheim, Germany) were cultivated at 37 °C in a humidified atmosphere of 5% CO_2_ in Dulbecco’s Modified Eagle’s Medium (DMEM) supplemented with a 10% fetal bovine serum (FBS, Gibco, NY, USA) and 1% penicillin/streptomycin. Cell viability was estimated by using the MTT assay as previously described [27]. In short, HaCaT cells were seeded in 96-well plates (10^4^ cells/well) and incubated with the samples diluted to achieve the desired concentrations of *Tempranillo* extract (0.1, 1, and 10 µg/mL). After 24 h, the cells were incubated with the MTT solution for 3 h. The formed purple formazan crystals were dissolved in dimethyl sulfoxide (DMSO) and the absorbance was read at 590 nm.

The cellular ROS levels were determined with the 2′,7′-dichlorofuorescein diacetate (DCFH-DA) method [27]. HaCaT cells were incubated with the samples diluted to achieve the desired concentrations of the extract (0.1, 1, and 10 µg/mL) for 24 h. Then, the cells were incubated with 10 µM of DCFH-DA for 30 min. After the incubation, 1 mM of H_2_O_2_ was added to each well, and the fluorescence intensity of ROS-oxidized 2′,7′-dichlorofuorescein (DCF) was measured at 485/530 nm (excitation/emission wavelengths), recording data every 5 min for 60 min.

### 2.8. Statistical Analysis

The results were expressed as the mean ± the standard deviation (SD). Student’s *t*-test was performed to substantiate differences between groups. For intracellular antioxidant activity data, a two-way analysis of variance (ANOVA) was performed, followed by Tukey’s test. Differences were considered statistically significant for *p* values below 0.05.

## 3. Results and Discussion

### 3.1. Phenolic Compounds in Grape Pomace Extract

The *Tempranillo* grape pomace extract (TE) was obtained as a purple paste following a green extraction procedure. The yield of the extraction was 38.7%. Considering previous research [28], anthocyanins are the major components in red grape pomace, while the other components have been reported in smaller amounts. For this reason, in this study, only the former were quantified. The phenolic profile of the extract was characterized by using UHPLC-DAD-ESI-QToF/MS, and phenolic compounds were identified based on their retention times, UV-visible spectra and MS data (Table 1). The anthocyanins in the TE were determined by using HPLC-DAD-ESI-QqQ/MS. (Table 2).

Fourteen flavan-3-ols, monomers, and trimers of (-)-epicatechin and (+)-catechin; 10 flavonols, derivatives of quercetin and kaempferol; 1 hydroxycinnamic acid, a derivative of *p*-coumaric acid; and 13 anthocyanins, 3-*O*-glycosides of malvidin, petunidin, cyanidin, peonidin, and delphinidin being the major components, were identified in the extract. These findings were in accordance with previously reported work, where anthocyanins were also the main compounds found in red grape skin extracts, and among them, Mv-3-*O*-glc was the most important [29,30]. As for the other compounds, the most abundant were reported to be phenolic acids, flavan-3-ols, and flavonols [31]. In addition, oligomers and polymers of flavan-3-ols, such as (-)-epicatechin and (+)-catechin, have also been found in grape seeds [32].

### 3.2. Vesicle Design and Characterization

This study aimed to develop a nanoformulation of the *Tempranillo* grape pomace extract (TE). In particular, we examined whether transfersomes would allow the production of a safe and stable formulation to be applied on the skin as well as enhance the bioactivity of TE constituents, providing protection from oxidative damage at the cellular level.

To find an optimal nanoformulation for TE, a pre-formulation study involving the evaluation of multiple candidates against selected endpoints was carried out. The study explored several processing conditions, such as type and concentration of phospholipid, phospholipid:edge activator (Tween 80) ratio, and duration of sonication, to identify the lead candidate with optimal features (i.e., a small size, a high entrapment efficiency, and physical stability).

Furthermore, to evaluate the impact of the extract and the edge activator, TE transfersomes were compared with empty transfersomes alongside conventional empty and TE liposomes.

The light scattering results, as reported in Table 3, showed that the empty liposomes were approximately 130 nm in diameter, slightly polydisperse, and negatively charged. The loading of the extract significantly increased the average size (ca. 150 nm) and the polydispersity (P.I. 0.6). The modification of the formulation with the addition of Tween 80 resulted in a marked improvement in the above features. Both the empty and TE transfersomes were smaller, around 100 nm in diameter. These vesicles were also characterized by a good homogeneity (P.I. 0.29), and they maintained negative zeta potential values. These results pointed to the crucial role of Tween 80. It is reasonable to assume that the presence of Tween 80 promoted a better arrangement of the phospholipids and a better solubilization and distribution of the extract constituents within the vesicles. As a result, the transfersomes featured better physico-chemical and technological characteristics, such as size, homogeneity, storage stability (monitored for two months), and entrapment efficiency. The latter, calculated based on the amount of main anthocyanins (Delphinidin-3-*O*-glucoside, Mv-3-*O*-glc, and Peonidin-3-*O*-(6-*p*-coumaroyl)-glucoside+Malvidin-3-*O*-(6-*p*-coumaroyl)-glucoside) detected in the TE, was 66 ± 9%.

**Figure 2:**
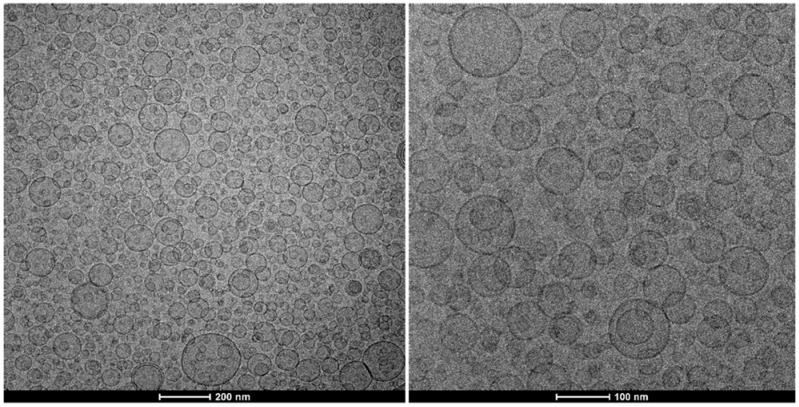
Cryo-TEM images of *Tempranillo* extract transfersomes. Two magnifications are shown: 29,000× (**left**) and 62,000× (**right**).

The formation of vesicular structures of small sizes was confirmed by performing a cryo-TEM observation. Figure 2 shows spherical, unilamellar vesicles of ca. 100 nm in diameter, which align with the light scattering data.

### 3.3. Antioxidant Activity of Grape Pomace Extract

The phenolic content of the TE was estimated using a Folin–Ciocalteu assay. The analysis performed on a TE methanolic solution gave a GAE value of 257 ± 36 µg/mL. The analysis of the TE vesicle formulations, however, uncovered lower values (180 ± 34 µg/mL). This was very likely due to the procedure employed: it did not involve the use of organic solvents and, apparently, the sonication step that we added was not effective enough to disrupt the vesicles and free the whole content. The antioxidant activity of the TE formulations was estimated as a function of their radical scavenging ability and ferric reducing ability. The TE methanolic solution scavenged the DPPH radical almost completely (AA 81 ± 2%), corresponding to 287 ± 18 μg/mL of Trolox equivalents. This was due to the well-known antioxidant power of the compounds identified in the extract. It should be noted that the antioxidant activity of TE in the transfersomes was slightly higher (AA 85 ± 3%; *p* < 0.01), corresponding to 308 ± 9 μg/mL of Trolox equivalents, due to the contribution of the vesicle carrier. Indeed, given the presence of phosphatidylcholine, the empty vesicles possessed slight antioxidant activity themselves (AA 38 ± 3%).

The results of the FRAP assay followed the same trend. The TE transfersomes showed a reducing power as strong as that of the free extract, which corresponded to 1011 ± 65 µg/mL of ferrous equivalents. Ultimately, these findings demonstrated that the formulation in the transfersomes preserved the intrinsic properties of the TE.

### 3.4. Cell Viability and Intracellular ROS Inhibition

**Figure 3:**
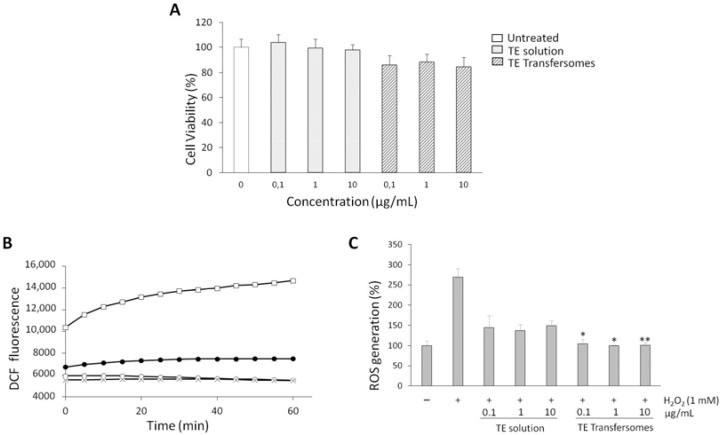
(**A**) Effects of *Tempranillo* extract (TE), in solution and in transfersomes, on HaCaT cell viability. (**B**) ROS levels (expressed as DCF fluorescence) in HaCaT cells pre-treated with *Tempranillo* extract (TE), in solution and in transfersomes (extract concentration: 1 µg/mL), and incubated with 1 mM of H_2_O_2_ for 60 min. (○): Untreated cells; (□): 1 mM H_2_O_2_; (●): TE + H_2_O_2_; (x): TE transfersomes + H_2_O_2_. (**C**) Effects of *Tempranillo* extract (TE), in solution and in transfersomes, on ROS production in HaCaT cells after a 1 h treatment with 1 mM of H_2_O_2_. Means ± SDs of three independent experiments, each performed in triplicate, are shown. Asterisks (*) indicate statistical difference between TE transfersomes and TE solution at each concentration: * *p* < 0.05, ** *p* < 0.005.

We examined whether TE transfersomes exert an antioxidant effect by inhibiting H_2_O_2_-induced ROS generation in cells. First, we assessed the cytocompatibility of free TE and TE transfersomes by evaluating their effects on the viability of human keratinocytes. After 24 h of exposure of the cells to various concentrations of the samples, the viability was examined using an MTT test. The results indicated that none of the samples were cytotoxic (Figure 3A). Indeed, cell viability remained around 85% when the highest concentration of TE transfersomes was tested, without statistically significant differences vs. untreated control cells.

To investigate the protective effects of TE transfersomes against oxidative stress, intracellular ROS levels were estimated using DCFH-DA. DCFH-DA is a non-fluorescent probe that is hydrolyzed to DCFH by intracellular esterases. The oxidation of DCFH by ROS leads to the formation of DCF with an increase in fluorescence intensity [33]. Figure 3B shows the increase in fluorescence due to the exposure to H_2_O_2_, as well as how it decreased as a function of time upon the application of TE to the solution and the transfersomes (TE 1 µg/mL). The figure clearly shows that the TE transfersomes restored basal ROS levels, since the curve for the vesicle formulation was essentially superimposed on that of untreated cells (i.e., not exposed to H_2_O_2_). As shown in Figure 3C, the exposure to H_2_O_2_ significantly increased the ROS levels in the HaCaT cells, as expected. Both the TE in the solution and the TE transfersomes succeeded in reducing the ROS levels already at a concentration of 0.1 μg/mL. However, the TE transfersomes reduced H_2_O_2_-induced ROS production with a statistically significant effect with respect to the TE solution (Figure 3C). These findings suggested the incorporation of TE results in a greater antioxidant effect in a cellular system.

## 4. Conclusions

The results of the present work showed that an extract rich in antioxidants can be obtained from food processing residues (e.g., grape pomaces) using a green extraction method. Furthermore, the bioactive constituents of the extract can be formulated in a vesicle system. Transfersomes were demonstrated to be able to incorporate, protect, and deliver *Tempranillo* grape pomace extract to cells. The in vitro results highlighted the ability of the vesicle formulation to counteract ROS overproduction, enhancing the effect of the extract in the cells. Therefore, the proposed formulation offers great promise as a treatment option for skin conditions that involve oxidative stress.

## Figures and Tables

**Table 1:** Identification of phenolic compounds in *Tempranillo* grape pomace extract determined by using UHPLC-DAD-ESI-QToF/MS analysis.

#	Compound	t_R_ (min)	DAD UV-Visible Bands (nm)	m/z [M + H]^+^	m/z [M − H]^−^
	**Flavan-3-ols**				
1	((Epi)catechin)_3_ (1) ^1^	3.29	283	867.199	865.199
2	Procyanidin B I	5.54	280	579.151	577.135
3	((Epi)catechin)_3_ (2) ^1^	5.73	283	867.213	865.199
4	Procyanidin B II	6.44	280	579.150	577.136
5	Catechin ^2^	7.53	278	291.087	289.072
6	((Epi)catechin)_3_ (3) ^1,2^	7.60	283	867.212	865.199
7	Procyanidin B III	8.19	280	579.150	577.135
8	((Epi)catechin)_3_ (4) ^1^	8.65	283	867.214	865.199
9	Procyanidin B IV	12.10	280	579.151	577.135
10	((Epi)catechin)_3_ (5) ^1^	12.91	283	867.214	865.199
11	Epicatechin	16.31	278	291.087	289.072
12	((Epi)catechin)_3_ (6) ^1^	17.39	283	867.216	865.199
13	Procyanidin B gallate	19.44	280	731.160	729.140
14	((Epi)catechin)_3_ (7) ^1^	20.53	283	867.216	865.199
	**Flavonols**				
15	Quercetin-hexosyl-hexoside-1	23.80	264, 344	627.157	625.137
16	Quercetin-hexosyl-hexoside-2	25.20	264, 344	627.156	625.140
17	Quercetin-3-*O*-galactoside	27.64	255,353	n.d. ^3^	463.082
18	Quercetin-3-*O*-glucuronide	27.89	255, 352	479.082	477.067
19	Quercetin-3-*O*-glucoside	28.38	255, 352	n.d. ^3^	463.092
20	Kaempferol-3-*O*-galactoside	30.21	265, 345	449.108	447.093
21	Kaempferol-3-*O*-glucuronide	31.00	265, 345	463.088	461.070
22	Kaempferol-3-*O*-glucoside	31.51	265, 348	449.108	447.093
23	Isorhamnetin-3-*O*-galactoside	31.51	254, 352	479.119	477.103
24	Isorhamnetin-3-*O*-glucoside	32.41	254, 352	479.119	477.104
	**Hydroxycinnamic acids**				
25	*p*-coumaroyl hexoside	10.46	313	n.d. ^3^	325.092

^1^ (Epi)catechin: (+)-catechin or (-)-epicatechin, unknown isomer. ^2^ Coeluting compounds. ^3^ N.d.: not detected.

**Table 2:** Determination of anthocyanins in *Tempranillo* grape pomace extract determined by using HPLC-DAD-ESI-QqQ/MS analysis.

#	Compound	DAD UV-Visible Bands (nm)	t_R_ (min)	m/z [M]^+^	m/z [Y_0_]^+^	Conc. (µg Mv-3-*O*-glc Equivalents/g Dry Pomace)
1	Delphinidin-3-*O*-glucoside	276, 526	8.97	465	303	235.02
2	Cyanidin-3-*O*-glucoside	279, 519	12.63	449	287	49.39
3	Petunidin-3-*O*-glucoside	276, 526	14.48	479	317	201.99
4	Peonidin-3-*O*-glucoside	278, 519	19.45	463	301	118.26
5	Malvidin-3-*O*-glucoside	276, 526	21.57	493	331	585.04
6	Delphinidin-3-*O*-(6-*O*-acetyl)-glucoside	275, 529	28.23	507	303	5.25
7	Petunidin-3-*O*-(6-*O*-acetyl)-glucoside	273, 526	36.10	521	317	6.19
8	Peonidin-3-*O*-(6-*O*-acetyl)-glucoside	278, 526	39.43	505	301	<4.98 ^1^
9	Malvidin-3-*O*-(6-*O*-acetyl)-glucoside	279, 526	40.03	535	331	47.04
10	Malvidin-3-*O*-(6-*O*-caffeoyl)-glucoside	279, 543	41.88	655	331	10.81
11	Petunidin-3-*O*-(6-*p*-coumaroyl)-glucoside	279, 531	43.00	625	317	22.10
12	Peonidin-3-*O*-(6-*p*-coumaroyl)-glucoside ^2^	279, 531	45.88	609	301	134.44
13	Malvidin-3-*O*-(6-*p*-coumaroyl)-glucoside ^2^	281, 532	-	639	331	-

^1^ <5 µg Mv-3-*O*-glc equivalents/g pomace: below lower limit of calibration. ^2^ Coeluting compounds.

**Table 3:** Characteristics of TE transfersomes in comparison with TE liposomes, empty liposomes, and empty transfersomes: mean diameter, polydispersity index (P.I.), and zeta potential (ZP). The values are the means ± the SDs (*n* > 6). ** TE transfersomes vs. TE liposomes: ** *p* < 0.01; ^••^ TE transfersomes vs. empty transfersomes: ^••^ *p* < 0.01; ^§§^ TE liposomes vs. empty liposomes: ^§§^ *p* < 0.01; ^#^ empty transfersomes vs. empty liposomes: ^#^ *p* < 0.05, ^##^ *p* < 0.01.

Formulation	MD (nm)	P.I.	ZP (mV)
TE transfersomes	** 105 ± 8	** 0.29 ± 0.03	**^••^ −9 ± 2
TE liposomes	^§§^ 155 ± 16	^§§^ 0.59 ± 0.04	^§§^ −4 ± 1
Empty transfersomes	^##^ 106 ± 17	^#^ 0.29 ± 0.02	^##^ −16 ± 2
Empty liposomes	128 ± 2	0.33 ± 0.03	−9 ± 2

## Data Availability

The data presented in this study are available within this article.
